# Cardiovascular Effects of Nickel in Ambient Air

**DOI:** 10.1289/ehp.9150

**Published:** 2006-07-20

**Authors:** Morton Lippmann, Kazuhiko Ito, Jing-Shiang Hwang, Polina Maciejczyk, Lung-Chi Chen

**Affiliations:** 1 New York University School of Medicine, Nelson Institute of Environmental Medicine, Tuxedo, New York, USA; 2 Institute of Statistical Science, Academia Sinica, Taipei, Taiwan

**Keywords:** atherosclerosis, cardiac function, cardiovascular mortality, concentrated airborne particles (CAPs), fine particulate matter, heart rate, heart rate variability, nickel, susceptible populations, vanadium

## Abstract

**Background:**

Fine particulate matter (FPM) in ambient air causes premature mortality due to cardiac disease in susceptible populations.

**Objective:**

Our objective in this study was to determine the most influential FPM components.

**Methods:**

A mouse model of atherosclerosis (ApoE^−/−^) was exposed to either filtered air or concentrated FPM (CAPs) in Tuxedo, New York (85 μg/m^3^ average, 6 hr/day, 5 days/week, for 6 months), and the FPM elemental composition was determined for each day. We also examined associations between PM components and mortality for two population studies: National Mortality and Morbidity Air Pollution Study (NMMAPS) and Hong Kong.

**Results:**

For the CAPs-exposed mice, the average of nickel was 43 ng/m^3^, but on 14 days, there were Ni peaks at ~ 175 ng/m^3^ and unusually low FPM and vanadium. For those days, back-trajectory analyses identified a remote Ni point source. Electrocardiographic measurements on CAPs-exposed and sham-exposed mice showed Ni to be significantly associated with acute changes in heart rate and its variability. In NMMAPS, daily mortality rates in the 60 cities with recent speciation data were significantly associated with average Ni and V, but not with other measured species. Also, the Hong Kong sulfur intervention produced sharp drops in sulfur dioxide, Ni, and V, but not other components, corresponding to the intervention-related reduction in cardiovascular and pulmonary mortality.

**Conclusions:**

Known biological mechanisms cannot account for the significant associations between Ni with the acute cardiac function changes in the mice or with cardiovascular mortality in people at low ambient air concentrations; therefore, further research is needed.

Exposures to levels of fine ambient particulate matter (FPM) near the current National Ambient Air Quality Standards have been associated with excess daily mortality and morbidity [U.S. Environmental Protection Agency (EPA) 2004, 2005]. However, the components of FPM responsible for these effects are still unknown. We have been studying the contributions of specific components of ambient air PM to cardiopulmonary and other health effects in humans and laboratory animals in recent years. Pope et al. (2002, 2004) documented that most excess annual mortality is associated with ambient air FPM, and is due to cardiac causes. This led us to develop the first practical experimental system to study the acute and cumulative effects of daily inhalation exposures to inertially concentrated ambient-air FPM (CAPs) in Sterling Forest (Tuxedo, NY), in apo-lipoprotein E-deficient (ApoE^−/−^) mice, an established mouse model of atherosclerosis, and in C57 mice, used as normal controls. The results of the first of these studies involving 5–6 months of warm-season daily exposures (5 days/week, 6 hr/day to an average CAPs concentration of 110 μg/m^3^) have been reported previously (Chen and Hwang 2005; Chen and Nadziejko 2005; Gunnison and Chen 2005; Hwang et al. 2005; Lippmann et al. 2005a, 2005b, 2005c; Maciejczyk and Chen 2005; Maciejczyk et al. 2005; Veronesi et al. 2005). These studies documented significant CAPs exposure–associated acute and chronic effects on cardiac function, increased amounts of and more invasive aortic plaque, and changes in brain cell distribution and in gene expression markers in the ApoE^−/−^ mice, as well as data on the effects of daily CAPs exposures on BEAS-2B human bronchial epithelial cells *in vitro* on nuclear factor kappa B (NFκB) activation. The effects on the C57 mice did not reach levels of statistical significance.

Most of the results of our subchronic CAPs exposure studies for the three winter months and for the six summer and fall months, also performed in Sterling Forest, have not yet been published. Results from our study for the six summer and fall months using our atherosclerotic mouse model (Sun et al. 2005) showed that CAPs (average, 85 μg/m^3^) enhanced atherogenesis in mice fed a high-fat diet, with accompanying increases in lipid content; enhanced vasoconstrictor responses to phenylephrine and serotonin challenge in the thoracic aorta; attenuated relaxation to the endothelium-dependent agonist against acetylcholine; and marked increases in macrophage infiltration, inducible isoform of nitric oxide synthase, generation of reactive oxygen species, and immunostaining for the protein nitration product 3-nitrotyrosine. In ApoE^−/−^ mice on a normal fat diet, some of these effects did not reach a level of statistical significance. In this article, we provide analyses of this subchronic CAPs inhalation study showing daily and long-term changes in cardiac function over the 6-month exposure period in ApoE^−/−^ mice on a high-fat diet.

Our published analyses of results of these subchronic CAPs inhalation studies in ApoE^−/−^ mice (Chen and Hwang 2005; Chen and Nadziejko 2005; Gunnison and Chen 2005; Hwang et al. 2005; Lippmann et al. 2005a, 2005b, 2005c; Maciejczyk and Chen 2005; Maciejczyk et al. 2005; Sun et al. 2005; Veronesi et al. 2005) have already demonstrated a new and higher level of biological plausibility for the excess annual mortality due to cardiac causes that are associated with chronic exposure to ambient air FPM in the American Cancer Society cohort (Pope et al. 1995, 2002, 2004), the Six Cities Study (Dockery et al. 1993; Laden et al. 2006), and the Adventist Health Study on the Health Effects of Smog (AHSMOG) (Chen et al. 2005). Our analyses have also demonstrated that 30 hr/week exposures to CAPs at concentrations near 100 μg/m^3^ (inhaled at Sterling Forest, which is located in a large state park and has no significant local sources of air pollution) can produce the kinds of adverse health effects that afflict susceptible elements of the general population (Chen and Hwang 2005; Chen and Nadziejko 2005; Gunnison and Chen 2005; Hwang et al. 2005; Lippmann et al. 2005a, 2005b, 2005c; Maciejczyk and Chen 2005; Maciejczyk et al. 2005; Sun et al. 2005; Veronesi et al. 2005). This kind of FPM, largely attributable to secondary aerosol, which arrives by long-range atmospheric transport from the southwest to the northwest of Sterling Forest, is inhaled by virtually all people downwind of the multiple upwind sources of precursor pollutants. Furthermore, we have demonstrated that our analyses of the elemental composition of the 6-hr filter samples, collected each day during each subchronic CAPs inhalation study, can inform us as to FPM components that affect health-related responses. Examples include the influence of residual oil combustion effluents on NFκB activation *in vitro* (Maciejczyk et al. 2005) and the influence, at different times of day, of several source-related components on cardiac function (Lippmann et al. 2005c).

During our analysis of the daily variations in cardiac function in the present 6-month (summer and fall) subchronic CAPs inhalation study, we noted the presence of a number of dramatic changes in cardiac function on certain days in the fall months. These observations led us to analyze the influence of daily variations in FPM component elemental concentrations on acute responses to ambient air FPM in terms of cardiac function in our mouse model of atherosclerosis. We found strong correlations with three metals (nickel, iron, and chromium) that generate reactive oxygen species (ROS) (Maciejczyk and Chen 2005). Because we were aware of suggestive evidence from research on the health benefits of a mandated switch to low sulfur fuels in Hong Kong in 1990 (Hedley et al. 2002) that were associated with substantial reductions in sulfur dioxide (Hedley et al. 2002) and vanadium and Ni (Hedley AJ, Chau PYK, Wong CM, unpublished data), we wondered if Ni may have been responsible for the notably high daily mortality associated with particulate matter < 10 μm in aerodynamic diameter (PM_10_) in New York City (NYC) (Dominici et al. 2003). The PM_10_ mortality coefficient for NYC in the National Mortality and Morbidity Air Pollution Study (NMMAPS) reanalysis [Johns Hopkins School of Public Health (JHSPH) 2003] was 3.8 times higher than the average. Ni, from residual oil combustion in oil-fired power plants and ocean-going ships in port, is contained in the FPM, which in NYC was 9.5 times higher than the average for 60 NMMAPS metropolitan statistical areas (MSAs) with available speciation data for 2000–2003.

These preliminary observations led us to undertake a more thorough examination of the associations between the average concentrations of metals in U.S. metropolitan areas and the NMMPS coefficients of daily mortality in these areas. Our analyses of the effects of metals concentrations on indices of acute health responses in both mice and humans are the primary subjects of this article.

## Methods

### Subchronic CAPs inhalation exposure study in ApoE^−/−^ mice

#### Animals

In this study we used 6-week-old male ApoE^−/−^ mice (Taconic Europe, Ry, Denmark). Animals were housed in single cages in an animal facility accredited by the Association for Assessment and Accreditation of Laboratory Animal Care. They were fed high-fat chow (Adjusted Calories Diet, TD 88137; Harlan, Indianapolis, IN, USA) for 2 weeks before being exposed to either CAPs or filtered air. Three weeks before the start of the exposure series, the mice were implanted with electrocardiograph (ECG) transmitters (TA10ETA-F20; Data Sciences, St. Paul, MN, USA). Ten-second ECG, heart rate (HR), activity, and body temperature data were sampled every 5 min throughout the experiment except during brief periods to transport animals between animal housing and the exposure facility. The environmental temperature and lighting schedule were automatically monitored every 5 min throughout the experiment. Body weights were recorded weekly, and physical health conditions were observed and entered into the database by experienced laboratory technicians. The Committees on Use and Care of Animals of the New York University approved all experimental procedures. All mice used in this research were treated humanely according to institutional guidelines, with due consideration for the alleviation of distress and discomfort.

#### FPM exposure

The experimental methods for the whole-body exposures of the mice to CAPs or filtered air in NYU’s A.J. Lanza Laboratory in Sterling Forest (Tuxedo, NY, USA) and for the daily 6-hr monitoring of the ambient air mass and elemental composition have been previously described (Maciejczyk et al. 2005). Briefly, six mice were exposed to CAPs at 10 times ambient concentrations for 6 hr/day, 5 days/week, for 6 months. Six control mice were sham exposed to an identical protocol, except that a high-efficiency particulate air (HEPA) filter was positioned at the inlet to remove all of the FPM in the filtered air stream. Exposures began on 21 July 2004 and were stopped on 12 January 2005. For the ambient FPM and for CAPs in the exposure chambers, particle samples were collected on Teflon filters (Gelman “Teflo,” 37 mm, 0.2 μm pore; Gelman Sciences, Ann Arbor, MI, USA) and weighed before and after sampling in a weighing room with controlled temperature and humidity. We used the weight gains to calculate the FPM exposure concentrations. Filter masses were measured on a microbalance (Model MT5; Mettler-Toledo Inc., Highstown, NJ, USA). Analyses for 34 elements were performed by nondestructive X-ray fluorescence spectroscopy (XRF) (model EX-6600–AF; Jordan Valley, Austin, TX, USA) using five secondary fluorescers (Si, Ti, Fe, Ge, and Mo), and spectral software XRF2000v3.1 (U.S. EPA and ManTech Environmental Technology, Inc., Research Triangle Park, NC, USA) according to the method described by Maciejczyk et al. (2005).

### Analysis of HR and HR variability in mice

Detailed methods for the analysis of the semicontinuous recordings of HR and HR variability (HRV) were described previously by Chen and Hwang (2005) and Hwang et al. (2005). In our previous study of HR and HRV (Chen and Hwang 2005; Hwang et al. 2005), we found that CAPs exposure most affected HR between 0130 hours and 0430 hours. To be consistent with our previous study, we used average HR and HRV [log SD of normal to normal beat intervals (logSDNN)] during the same period.

To estimate the effects of CAPs exposure on HR and log SNDD over the 6-month exposure period, we applied generalized additive models (GAMs) to fit the nonlinear trends of chronic effect changes and acute effects contributed by a specific component (Hastie and Tibshirani 1990). Specifically, *y_ijk_* represents the daily average of HR or logSDNN recorded during 0130–0430 hours in the *k* + first morning for the *j*th mouse in the *i*th group, where *i* = 1 represents the control group and *i* = 2 represents the exposure group. The model has the form


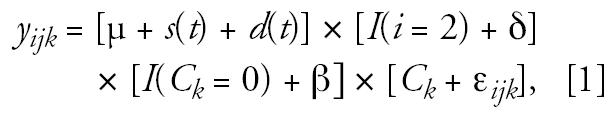


where *s*(*t*) and *d*(*t*) are smoothing spline functions with 4 degrees of freedom, *I* is an indicator function, *C_k_* is the exposure concentration in log scale of a specific component measured on the *k*th day, and ɛ*_ijk_* is an error term assumed to be normally distributed and independent with a constant variance. The parameter μ represents the overall mean of the health response variable, and δ represents the difference in response between nonexposure days and exposure days. The smoothing function *s*(*t*) was used to model the average health parameter changes for all the mice over the 6 months, whereas *d*(*t*) was used to model the health parameter differences between mice in the exposure group and the control group over the same time period. The estimates, with 95% confidence intervals, of the coefficient β for acute effects caused by specific components and the chronic effect function *d*(*t*) were obtained from the statistical package R (R Foundation for Statistical Computing 2006). The residual diagnostics were implemented to ensure that no serious violation of the model assumptions occurred.

### Time-series coefficients of daily mortality and concentrations of transition metals in NMMAPS

To examine the possible role of PM components (i.e., transition metals, ions, and crustal soil tracers) on the city-to-city variation of PM_10_ mortality risk estimates, we analyzed the association between the key FPM components from the FPM speciation network and the NMMAPS PM_10_ daily mortality risk estimates. The speciation data were obtained from the U.S. EPA Air Quality System (AQS) for the years 2000–2003 (U.S. EPA 2003). The NMMAPS PM_10_ mortality risk estimates (updated estimates using generalized linear modeling) for the 90 largest U.S. MSAs (for the time-series analysis that was conducted for 1987–1994) were obtained from the JHSPH Internet-based Health and Air Pollution Surveillance System (IHAPSS) website (JHSPH 2003). Although there were more than 40 FPM species, we focused on the 16 key components that were most closely associated with major source categories: aluminum, arsenic, Cr, copper, elemental carbon, Fe, manganese, Ni, nitrate, organic carbon, lead, selinium, silicon, sulfate, V, and zinc. First, for each FPM monitor, quarterly averages were computed from 24-hr average values (of at least every 6th-day schedule) when > 50% of scheduled samples were available. Second, an annual average for each FPM monitor was computed (but only when the four complete quarter averages were available). Third, the annual average values were then averaged across available monitors for each MSA. The resulting MSA-averaged FPM component values were then matched with the 60 NMMAPS MSAs that had FPM speciation data. Most of the annual speciation data were highly skewed. Therefore, we examined both raw and log-transformed data. The PM_10_ mortality risk estimates (expressed as percent excess deaths per 10-μg/m^3^ increase in PM_10_) were then regressed on each of the FPM components, with weights based on the SE of the PM_10_ risk estimates.

## Results

### Subchronic CAPs inhalation exposure in ApoE^−/−^ mice

#### Exposures

The concentrations of FPM and component elements both in ambient air and in the exposure chambers are summarized in Table 1.

**Figure 1:**
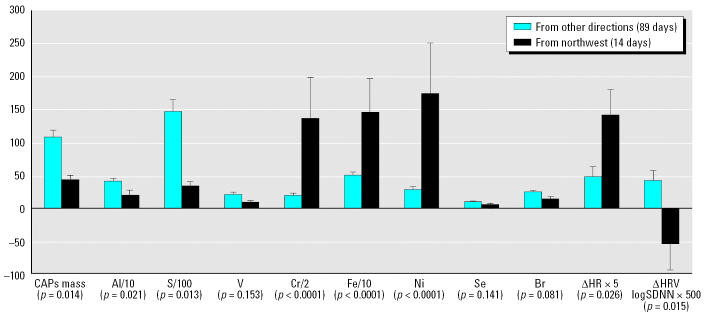
Elemental concentrations and HR and HRV (mean ± SE) for 14 days when winds were from the northwest and for the 89 days with winds from all other directions, and the differences in heart rates in ApoE^−/−^ mice exposed to CAPs and filtered air. CAPs concentrations are shown in μg/m^3^, elemental concentrations in ng/m^3^, HR in beats/min, and HRV (as logSDNN) in milliseconds.

We noted unusually high HRs during November and December 2004 that we had not seen in the previous subchronic mouse CAPs inhalation studies (Hwang et al. 2005); we then examined the associations of HR and HRV with the FPM mass and elemental concentrations that were measured each exposure day. We found the closest associations with Ni and Cr, and the days with high Ni and Cr had unusually low FPM mass concentrations. Figure 1 summarizes the differences between the 14 days with unusually elevated HRs and all of the other exposure days in terms of the exposure chamber concentrations of FPM, Al, S, V, Cr, Fe, Ni, Se, and bromine, along with the average difference in HR and HRV between the CAPs-exposed and air-sham–exposed ApoE^−/−^mice. Assuming that the Ni, Cr, and Fe were associated with SO_4_^2−^, they accounted for 12.4% of the FPM mass on those 14 days and only 1.5% on the other days.

#### Back trajectory analyses

**Figure 2:**
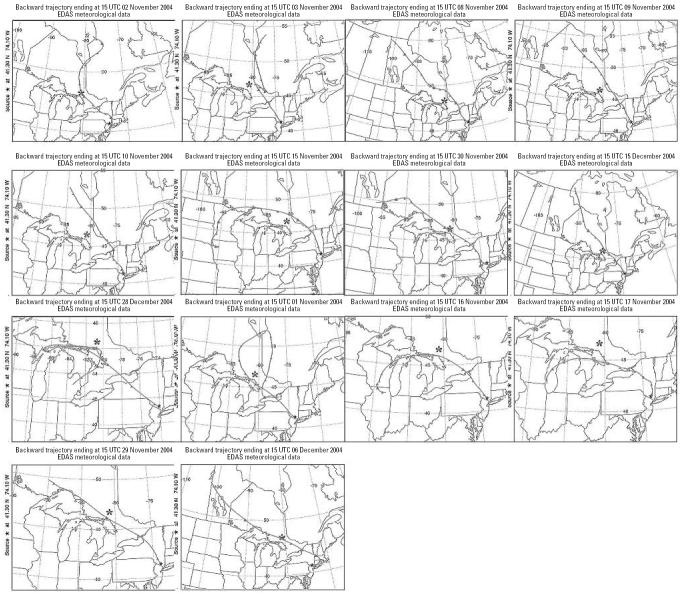
Back trajectories of all 14 days when winds were from the northwest based on the National Oceanic and Atmospheric Administration (NOAA) HYSPLIT model (NOAA 2006). Abbreviations: EDAS, Eta Data Assimilation System; UTC, coordinated universal time. *Location of the nickel smelter in Sudbury, Ontario, Canada.

We obtained back trajectory maps for the 14 days with the most notably elevated HRs, which were all associated with high-altitude winds from the northwest (Figure 2). The 72-hr back trajectories from Sterling Forest for this period appear to avoid population centers and industrial areas other than the Ni smelter near Sudbury, Ontario, Canada, which discharges its airborne effluents through a very tall smokestack.

**Figure 3:**
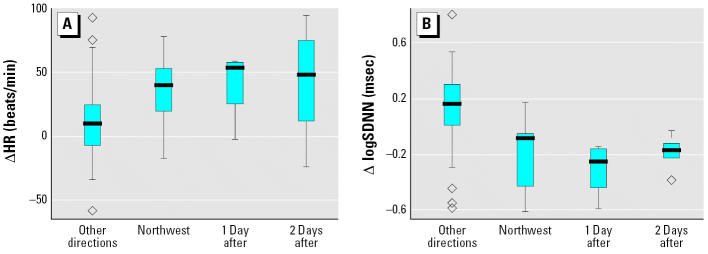
Differences in daily group–averaged HR (*A*) and HRV (logSDNN) (*B*) between mice exposed to CAPs and filtered air for the 89 days when winds were from other directions and for the 14 days when winds were from the northwest (and Ni, Cr, and Fe were elevated) with the corresponding data for 1 and 2 days after the days with wind from the northwest. Boxes represent 25th and 75th percentiles, lines within boxes indicate medians, error bars indicate 10th and 90th percentiles, and diamonds indicate outliers.

We also examined the lag structure for the temporal associations of the Cr, Fe, and Ni peaks and the differences in HR and HRV. Distributions of daily group–averaged HR and HRV differences between mice exposed to CAPs and filtered air, grouped by the 14 days when winds were from the northwest, all 89 exposure days when winds were from other directions, and days with 1 and 2 days lag of the northwest wind days. As shown in Figure 3A, we found that the HR elevation in mice exposed to CAPs lasted for at least 2 days. Using the *t*-test, we found that the HR elevations were significant for the current day (*p* = 0.016) but not significant for 1 day (*p* = 0.115) or 2 days (*p* = 0.228) later. The effect on HRV reduction also persisted for at least 2 days, and the differences were significant for all three days using the *t*-test (*p* < 0.017), as shown in Figure 3B.

**Figure 4:**
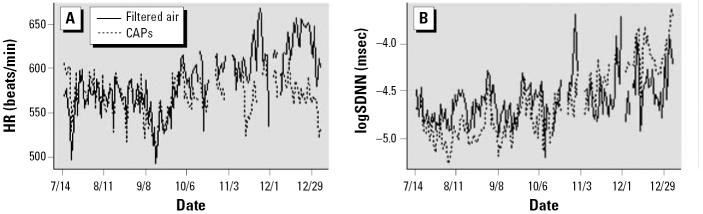
Daily group averaged HR (*A*) and HRV (logSDNN) (*B*) in mice exposed to CAPs or filtered air.

As shown in Figure 4, there were persistent changes in baseline HR and HRV, with the differences being most pronounced after the series of 14 days with unusually high Ni values.

The estimated acute effect coefficients for the four FPM components for HR tabulated in Table 2 were based on log concentrations for four FPM components. For Ni, it was 3.321, implying that the HR in the mice had an increase of approximately 7.6 and approximately 15 beats/min for an increase in exposure of 10 and 100 ng/m^3^ Ni, respectively. We also determined the coefficients for V, Cr, and Fe separately; however, the acute effects of V, Cr, and Fe fell short of statistical significance.

The estimated acute effect coefficients for the same four FPM components are tabulated in Table 3 for HRV, where only Ni produced a statistically significant effect. The estimated coefficient for Ni of 0.044 implies that SDNN had decreases of approximately 10% and approximately 20% for increases in exposure of 10 and 100 ng/m^3^ Ni, respectively.

### Time-series coefficients of daily mortality and average transition metals concentrations in the 60 NMMAPS MSAs

**Figure 5:**
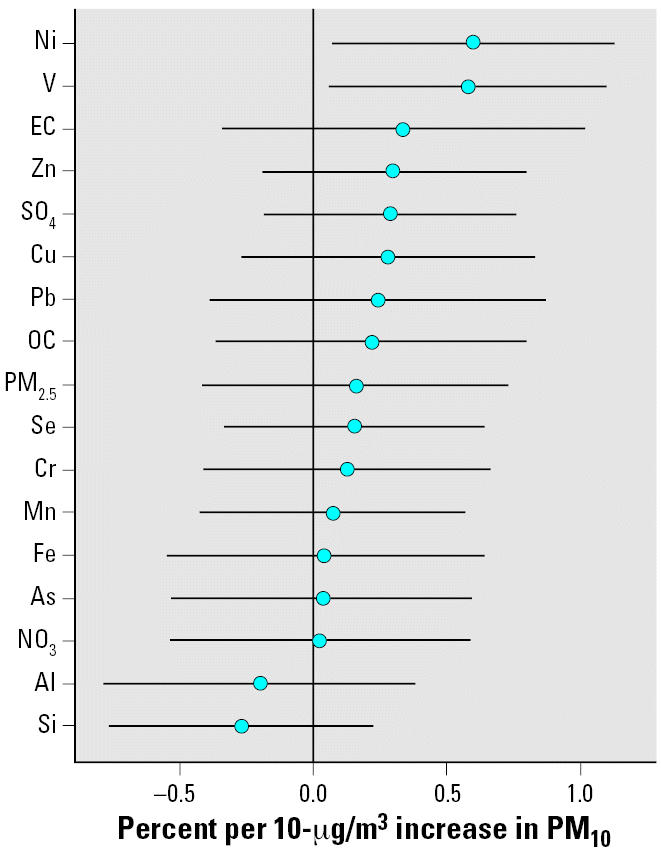
Differences in mortality risk coefficients shown as the 5th–95th percentile difference in concentrations of FPM and FPM components for the 60 NMMAP MSAs for which FPM speciation data were available. Abbreviations: EC, elemental carbon; OC, organic carbon.

For all of the FPM components we examined, the predictive power increased when the log-transformed variables were used. Figure 5 shows the resulting difference in the PM_10_ mortality risk estimates (in percent per 10-μg/m^3^ increase in PM_10_) shown as the 5th–95th percentile difference in the FPM component across the 60 NMMAPS MSAs for which speciation data were available. For example, for Ni and V, the PM_10_ risk coefficients were high (0.6) in the MSAs where Ni and V were significantly high (95th percentile) compared with the MSAs where Ni was low (5th percentile). These differences in magnitude were not small: the nationwide combined estimate for the 90 MSAs in the NMMAPS study was 0.21. Ni and V, which are most strongly associated with residual oil combustion effluent, showed the strongest predictions of the variation in PM_10_ risk estimates across the NMMAPS MSAs, followed by elevated but nonsignificant increases > 0.21 that were associated with elemental carbon, Zn, SO_4_^2−^, Cu, Pb, and organic carbon. The metals most closely associated with resuspended soil (i.e., Al and Si) had the lowest values, suggesting that they were unlikely to influence daily mortality. Thus, FPM components appear to explain some of the MSA-to-MSA variation in the NMMAPS PM_10_ daily mortality risk coefficients.

## Discussion

### Subchronic CAPs inhalation exposure in ApoE^−/−^ mice

The results shown in Figures 1, 3, and 4 are consistent with an acute effect of relatively low concentrations of Sudbury smelter effluent on cardiac function in the ApoE^−/−^ mouse model of atherosclerosis, a model that has increasingly been used as a model for humans with atherosclerosis. We found significant acute elevations in HR and significant reductions in HRV, both of which are generally considered to be indicators of cardiac stress.

Because the majority of the animal CAPs inhalation studies conducted by others in the past involved relatively few days of exposure (Campen et al. 2001; Muggenburg et al. 2003; Watkinson et al. 2000; Wellenius et al. 2002), there are no comparable published results. We are aware of only one previous study in which animals were exposed to CAPs for which compositional data were available. In that study, Wellenius et al. (2002) exposed dogs with vascular occluders around their left anterior descending artery to Boston, Massachusetts, CAPs at an average concentration of 285 μg/m^3^ for 6 hr/day over 3 or 4 days. After each CAPs exposure, there was a 5 min coronary artery occlusion. The CAPs exposures and occlusions were associated with peak ST-segment elevations that were significantly associated with the concentrations of Si (8.2 μg/m^3^, *p* = 0.003) and Pb (27 μg/m^3^, *p* < 0.05), but not with Ni (0.16 μg/m^3^). The quite different and apparently opposite influences of Ni and Si on cardiac function described by Wellenius et al. (2002) in dogs and the present study in mice may be caused by differences in animal models, the time of day that the functional measurements were made, the sedation and physical restraints used for the dogs, the possibility that the responses in mice were influenced by their ongoing repetitive daily CAPs exposures, and/or that the responses were influenced by other unrecorded variables.

Inhalation exposures to resuspended emission source particles that are enriched in transition metals such as Ni, V, and Fe have reported effects of FPM on cardiovascular function. For example, 3 mg/m^3^ Boston residual oil fly ash (ROFA) produced arrhythmias, ECG abnormalities, and decreases in HRV in a rat model of myocardial infarction (Wellenius et al. 2002). Similar exposure in beagle dogs had no effects on ECG but produced a trend toward decreased HR (Muggenburg et al. 2000). Increased arrhythmias, decreased HRs, and hypothermia in compromised animals were observed in monocrotaline-treated Sprague-Dawley rats exposed to 15 mg/m^3^ ROFA (Watkinson et al. 2000). The same concentration in spontaneously hypertensive (SH) rats caused cardiomyopathy, monocytic cell infiltration, and increased expression of cardiac cytokines interleukin-6 and transforming growth factor-β. The ROFA-exposed SH rats also showed increased ECG abnormalities compared with air-exposed SH rats.

In contrast, short-term inhalation of a single Ni compound did not produce any acute changes in cardiovascular function. Muggenburg et al. (2003) observed no significant effect of oral inhalation exposure to 50 μg/m^3^ of oxides or sulfates of Ni and V for 3 hr/day for 3 consecutive days in old dogs having preexisting cardiac abnormalities. However, in a different study, NiSO_4_^2−^ (> 1.2 mg/m^3^ for 6 hr/day for 4 days) caused delayed bradycardia, hypothermia, and arrhythmogenesis in rats (Campen et al. 2001). Similar exposure to V and SO_4_^2−^ failed to induce any significant change in HR or body temperature. In the same study, V appeared to enhance the cardiovascular effects of Ni.

### Time-series coefficients of daily mortality and concentrations of transition metals and other FPM components in the 60 NMMAPS MSAs

Our analysis of the 60 NMMAPS MSAs for which speciation data were available showed that the PM_10_ mortality risk estimates (in percent per 10-μg/m^3^ increase in PM_10_) were high (0.6) for Ni and V in the MSAs where Ni and V were significantly high (95th percentile), compared with the MSAs where Ni was low (5th percentile) (Figure 5). These results, however, should be interpreted with some caution because they rely on comparisons between PM_10_-associated daily mortality for 1987–1994 and FPM composition data for 2000–2003. With the exception of Al and Si, which are present mostly in coarse mode dust particles, and Fe, which is present in appreciable amounts in both the fine and coarse modes, the PM components in Figure 5 are found primarily in the FPM range, and their concentrations in PM_10_ and FPM would be similar in any given year. There was, however, almost certainly a significant temporal change in FPM composition between 1990 and 1995 due to a Clean Air Act–mandated 50% reduction in SO_2_ emissions from power plants (Clean Air Act Amendments 1990) The reduction in emissions of SO_2_ was almost certainly accompanied by some corresponding reductions in SO_4_^2−^, Se, and Ni from coal-fired power plants, introducing some exposure characterization error in the mortality coefficients used in Figure 5. Such exposure errors tend to reduce coefficients of response and their statistical significance.

There has been one previous population study whose overall results can be interpreted as being consistent with an effect of Ni and/or V on daily mortality. Hedley et al. documented large reductions in the concentrations of Ni and V, but not in other metals, in Hong Kong after 1 July 1990 as a result of a mandated switch to fossil fuels with low S content (Hedley AJ, Chau PYK, Wong CM, unpublished data). This group (Hedley et al. 2002) showed that the switch to low S fuels caused a simultaneous drop of about 50% in ambient air SO_2_, but no intervention-associated changes in the concentrations of other airborne criteria pollutants. The drop in SO_2_ was associated with prompt and persistent reductions in daily mortality of 2.2% overall, with about 2% for cardiac mortality, and about 4.5% for respiratory mortality.

When taken together, our findings of acute and chronic changes in HR and HRV in our mouse inhalation study (in which V concentrations were not elevated) and the results of the Hong Kong sulfur-in-fuel intervention study, which found a sudden and persistent drop in mortality associated with the sudden declines in the ambient air concentrations of Ni and V (Hedley AJ, Chau PYK, Wong CM, unpublished data), suggest that Ni was a more likely contributory factor than V on acute cardiac stress. In terms of the effects of long-term averages of daily mortality coefficients, our own analysis of coefficients for 60 NMMAPS MSAs in relation to FPM components indicated that both Ni and V were important determinants of response. These data suggest that other FPM exposure components may also play important roles, at least on days when the Sudbury smelter plume was not a major FPM source. Thus, we cannot rule out a role for V on cardiac-related mortality on days when it is present at higher concentrations.

### Biological plausibility of Ni to produce cardiovascular effects

Very few studies have investigated the mechanisms of cardiovascular effects of Ni. Ni ions have been reported to induce vasoconstriction and early afterdepolarization in isolated rat heart and canine coronary artery by enhancing Ca^2+^ influx into vascular smooth muscle cells (Golovko et al. 2003; Koller et al. 1982; Rubanyi and Kovach 1980). Plant et al. (2005) reported that the release of Ni ions from Ni–Ti alloy of intravascular stents by mechanical polishing caused increased oxidative stress in human umbilical vein endothelial cells. At the molecular level, Ni has been found to be involved in many signaling pathways and some of these pathways are critical in the development of cardiovascular disease. For example, Ni turns off the expression of thrombospodin 1 (a regulator of angiogenesis) and activates hypoxia-inducible factor 1 (an important factor in regulating cellular oxygen concentration) and NFκB (with subsequent up-regulation of the intracellular, vascular, and endothelial adhesion molecules ICAM-1, VCAM-1, and E-selectin, respectively) [see the review by Denkhaus and Salnikow (2002)]. Thus, Ni exposure may play a key role in leukocyte recruitment in the vasculature leading to vascular inflammation and dysfunction, resulting in enhanced progression of atherosclerosis seen in mice exposed to FPM CAPs (Sun et al. 2005). Advanced atherosclerosis could reduce coronary blood flow leading to dysrythmia and myocardial ischemia, which may be reflected in changes in HR and HRV in mice and increased risk of mortality in humans described above.

### Other health effects associated with high-concentration Ni exposures

To the extent that airborne Ni in ambient air has been considered a risk factor for human health, it has been in the context of carcinogenesis [World Health Organization (WHO) 2000]. Regarding occupational settings where airborne concentrations have been very high, Costa (2000) summarized evidence in the literature for excess respiratory tract cancer in Ni workers in studies by Cuckle et al. (1980), Cox et al. (1981), Doll et al. (1977), Enterline and Marsh (1982), Godbold and Tompkin (1979), the International Agency for Research on Cancer (IARC 1976, 1990), and Magnus et al. (1982). In terms of other health effects in occupational settings, McConnell et al. (1973) reported asthma exacerbation associated with the inhalation of Ni-containing mists, and Costa (2000) summarized evidence for dermal and respiratory allergic reactions. To date, there have been no studies of working populations that have related Ni exposure to cardiovascular disease.

### Does Ni cause cardiac mortality associated with FPM?

The results of our analyses of the associations between annual average FPM component concentrations and average daily mortality coefficients in 60 NMMAPS MSAs (Figure 5) are broadly consistent with the Hong Kong experience (Hedley et al 2002; Hedley AJ, Chau PYK, Wong CM, unpublished data). In both populations, the concentrations of Ni and V in ambient air were associated with significant differences in mortality rates, whereas all other measured PM components were not. Both Ni and V are present at relatively high concentrations in residual oil combustion effluents from oil-fired power plants and/or ocean-going ship boilers consuming high-S fuel when operating in or near port cities. For example, in 2002, 93% of the U.S. emissions of Ni were in states that border the Atlantic and Gulf Coasts and in California, and 3% was from states that border the Great Lakes, leaving only 4% for the other 25 states (Energy Information Administration 2006). There are similar percentages for V. Thus, residual oil combustion effluents, as a source-related mixture, or the Ni and/or the V in that mixture, may be responsible for a disproportionate contribution to excess FPM-associated mortality in coastal cities. However, these results alone do not indicate the relative contributions of Ni and V to the overall mortality impact or whether they have differential effects on mortality due to cardiovascular and/or respiratory causes.

The results from our 6-month (summer and fall) subchronic CAPs inhalation study in a mouse model of atherosclerosis, (illustrated in Figures 1 and 3) indicate that inhalation exposure to Ni, compared with V, is a more likely causal factor for the exacerbation of cardiac disease. On the other hand, V and S (emitted from residual oil combustion, whether present as SO_2_ or as acidic aerosol) are likely to contribute to the exacerbation of respiratory disease outcomes such as the drop in bronchial hyperreactivity in Hong Kong after the sulfur-in-fuel intervention (Wong et al. 1998) and the associated drop in respiratory mortality. In contrast to Ni, V is capable of disrupting protein phosphorylation and activation of the three major branches of the mitogen-activated protein kinase signaling pathways (extracellular signal–regulated kinases, c-*Jun* amino-terminal kinases, and P38) in human airway epithelial cells (Ghio and Samet 1999; Samet et al. 1997) and may be largely responsible for the pulmonary toxicity of ROFA, which induces a rapid and marked increase in protein tyrosine phosphate accumulation in human airway epithelial cells.

Associations between ambient air Ni and cardiovascular disease have only recently become a research issue because of the presence of the appreciable Ni content in ROFA and associations between ROFA exposures and cardiac function in animals *in vivo* and tissues *in vitro*, and in our own findings in our first 6-month study of NFκB activation *in vitro* by the 2% of the CAPs that was attributable to residual oil combustion (Maciejczyk et al. 2005).

The few laboratory-based studies of Ni cardiovascular toxicity carried out in recent years (Campen et al. 2001; Muggenburg et al. 2003) have involved exposures at concentrations well above those considered to be relevant to ambient air exposures. Thus, the results of the present study represent a significant increment to our knowledge of the likely risks to human health of exposures to Ni in ambient air and especially to cardiovascular risks. The statistically significant short-term cardiac function changes in mice are associated with 6-hr average concentrations of Ni < 200 ng/m^3^. The effects associated with reduced mortality in humans in the NMMAPS study are associated with longer-term Ni concentrations extending from 19 ng/m^3^ in NYC down to a national average of 1.9 ng/m^3^.

If Ni inhalation at current ambient air concentrations does appreciably affect cardiac function and mortality in humans, why has it not previously been recognized? One reason may be that the increment in cardiovascular mortality produced by Ni is a relatively small part of the very large cardiovascular mortality. Also, the statistically significant transient and progressive changes that we have seen in cardiovascular function in our mice are relatively subtle, require advanced analytical techniques for their detection, and are unlikely to be detected in the types of short-term exposure studies that have previously been undertaken in laboratory animals.

## Conclusions

Much of the remaining skepticism concerning the biological plausibility of the premature mortality and increased morbidity associated with ambient air FPM has been due to the paucity of exposure–response data in laboratory studies involving FPM inhalation and the heretofore seemingly impossible task of identifying any specific causal components. The subchronic CAPs inhalation studies that we have performed in our laboratory at Sterling Forest have begun to establish such plausibility (Lippmann et al. 2005b) and have developed a mechanistic base for the initiation and progression of effects attributable the long-range transported aerosol in the northeastern United States (Sun et al. 2005). The chance occurrence of a series of days during our 6-month subchronic mouse CAPs inhalation study, in which northwest winds with low FPM concentrations but with greatly elevated concentrations of Ni attributable to the Ni smelter at Sudbury, gave us the opportunity to identify Ni as an FPM component of particular relevance to cardiac function, as presented in this article. We also had the opportunity to seek other available data that could be used to determine whether Ni was particularly influential in producing cardiovascular responses in human populations. We did find such evidence in our reexamination of the NMMAPS daily mortality data in conjunction with the newly available FPM speciation data, and with our further examination of the data available from the Hong Kong sulfur-in-fuel intervention study (Hedley et al. 2002). The consistency in all of these analyses and reexaminations of available data lead us to conclude *a*) that Ni is a particularly influential component of ambient FPM in terms of cardiac responses to the inhalation of ambient air FPM; and *b*) that further research is needed on the specific influences of both Ni and V, which are both generally most closely associated with residual oil combustion effluents, on both acute and chronic respiratory and cardiovascular health effects.

In terms of environmental relevance, it is important to recognize that the peak Ni concentrations in the CAPs were only approximately 175 ng/m^3^ on the peak Ni exposure days and only 26 ng/m^3^ on the 89 other days; there were no pronounced peaks for V (average, ~ 17 ng/m^3^). Thus, Ni appears to be the component most likely to cause acute cardiac responses. The long-term average ambient air level of Ni in the United States is 1.9 ng/m^3^, and the highest, in NYC, is 19 ng/m^3^. Biological mechanisms that account for the significant associations between Ni and the progression of cardiovascular disease in mice or cardiovascular mortality in people exposed at low, environmentally relevant, ambient air concentrations are unknown; these mechanisms warrant further research *in vivo* and *in vitro*.

## Figures and Tables

**Table 1:** Concentrations (mean ± SE) of selected elements in FPM CAPs from Sterling Forest (SF) and FPM from NYC and the United States.

Sample source	PM_2.5_ (μg/m^3^)	S (μg/m^3^)	Al (μg/m^3^)	V (ng/m^3^)	Ni (ng/m^3^)	Se (ng/m^3^)
Overall SF FPM CAPs	85.6 ± 8.2	11.32 ± 1.37	354.6 ± 28.7	17.3 ± 2.4	43.15 ± 10.1	8.7 ± 0.9
SF CAPs (14 days with winds from northwest)	43.7 ± 6.1	3.43 ± 0.56	199.1 ± 73.5	9.2 ± 2.2	174.0 ± 77.0	5.5 ± 1.9
SF CAPs (other 89 days)	91.0 ± 9.0	12.3 ± 1.5	374.6 ± 30.5	18.4 ± 2.7	26.3 ± 4.1	9.1 ± 1.0
NYC FPM^a^	15.7 ± 0.7	1.4 ± 0.1	16.5 ± 2.9	6.1 ± 0.3	19.0 ± 1.1	1.3 ± 0.1
National FPM^b^	14.4 ± 0.5	1.1 ± 0.1	33.4 ± 4.0	1.9 ± 0.2	1.9 ± 0.4	1.2 ± 0.1

a Mean values from three monitors in NYC during 2000–2002 (*n* = 202 days).

b Average values of annual mean across 60 MSAs.

**Table 2:** Summary of estimates of coefficient β for acute effect of four metals on HR in the GAM for single FPM components of interest.

Component	Estimate	SE	*p*-Value
Ni	3.321	1.628	0.041
V	3.273	2.006	0.103
Cr	2.916	1.787	0.103
Fe	2.997	2.412	0.214

**Table 3:** Summary of estimates of coefficient β for acute effect of four metals on logSDNN in the GAM for single FPM components of interest.

Component	Estimate	SE	*p*-Value
Ni	0.044	0.016	0.005
V	0.024	0.019	0.222
Cr	0.024	0.018	0.179
Fe	0.042	0.023	0.070
