# Erratum

**Published:** 2007-06

In Figure 4 of Lippmann et al. [ Environ Health Perspect 114:1662–1669 (2006)], the key was correct in the original manuscript published online but was incorrect in the final version. The dashed lines should indicate filtered air, and the solid lines should denote CAPS. The corrected figure appears below.

*EHP* regrets the error.

## Figures and Tables

**Figure 4:**
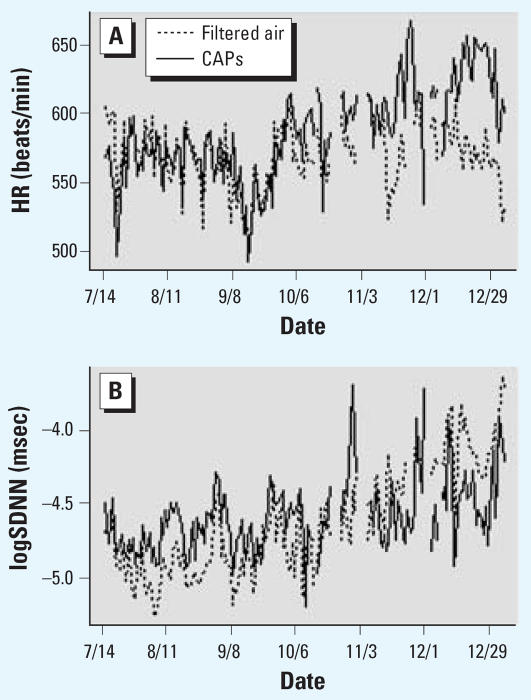
Daily group averaged HR (*A*) and HRV (logSDNN) (*B*) in mice exposed to CAPs or filtered air.

